# Advances in Diagnosis and Treatment of Biliopancreatic Diseases

**DOI:** 10.1155/2012/421969

**Published:** 2012-05-16

**Authors:** Fauze Maluf-Filho, Jose G. de la Mora Levy, Carlos G. Micames

**Affiliations:** ^1^Gastrointestinal Endoscopy Unit, Department of Gastroenterology, Instituto do Cancer do Estado de São Paulo (ICESP), São Paulo University, São Paulo, SP, Brazil; ^2^Endoscopy Unit, Instituto Nacional de Cancerología, Mexico City, Mexico; ^3^Hospital Bella Vista, University of Puerto Rico, Mayaguez, Puerto Rico, USA

Management of biliopancreatic diseases has seen great progress in recent years. Innovations in endoscopy have allowed gastroenterologists to assist in the care of many conditions of the biliary tree and pancreas, which were previously only managed surgically. Outcomes for many of these endoscopic interventions are comparable to their surgical counterpart. However, endoscopists taking care of these patients should be well aware of the limitations of their procedures and the advantages of surgery for certain patients. 

This special issue will address some of the main challenges faced today by physicians taking care of patients with pancreatic and biliary diseases. Endosonography, or endoscopic ultrasound (EUS), which previously was only considered a diagnostic tool, has now emerged as a useful therapeutic technique. In contrast, endoscopic retrograde cholangiopancreatography, or ERCP, has now become mainly a therapeutic tool. Some ERCP techniques have been adopted by endosonographers and allowed interventions for draining bile ducts and peripancreatic fluid collections, previously considered outside the realm of endoscopy. Despite the advances in outcomes and minimal invasiveness of EUS and ERCP-guided interventions, several risks and complications still remain. Pancreatitis is a well-recognized complication of ERCP with a significant incidence that has not been reduced greatly throughout recent years. Numerous studies have looked at ways of decreasing the risk of this dreadful complication. This special issue will discuss and review the problem of post-ERCP pancreatitis, a topic that raises a high level of concern for many who do this procedure, and ways to reduce its risk, especially in patients considered at high risk for developing this complication.

Incidental pancreatic cysts continue to pose a diagnostic dilemma for gastroenterologists and surgeons alike. Despite the ability of safely obtaining a tissue sample during EUS examination, the utility of cytopathology in determining the exact nature of these lesions remains limited. More recently, DNA analysis of fluid obtained during aspiration has shown promise in improving the diagnostic accuracy of EUS-FNA. However, the effect of these newer tests in terms of deciding when to observe versus surgically resect in many of these incidental cysts remains to be proven. Pancreatologists agree that a reliable method for determining the nature and prognosis of pancreatic cystic neoplasms is required for improving the management strategy in asymptomatic patients. 

Abdominal pain is a cardinal symptom of chronic pancreatitis. The effectiveness of medical therapy, in the form of pancreatic enzyme supplementation or octreotide injections, is dismal. Pain relief can certainly be obtained with the use of narcotic medications, but at the cost of significant side effects, such as constipation, sedation, and drug dependence. Endoscopic alternatives for improving pain in chronic pancreatitis include those guided by ERCP, such as intraductal stone removal, stricture dilation, or drainage procedures, and EUS-guided celiac plexus block. This special issue will review the ERCP-guided interventions and discuss the efficacy of EUS-guided celiac plexus neurolysis for the treatment of pain in patients with pancreatic cancer. 

As advances in endoscopy and surgical innovations are introduced into the field of biliary and pancreatic diseases, many diseases previously requiring complicated surgeries with prolonged recovery are now being managed by minimally invasive techniques. Many of these procedures have a reduced risk when compared to their surgical alternative. However, long-term efficacy and patient risk factors need close consideration when deciding between different management alternatives. Many, if not all, biliary and pancreatic disorders require a close interaction between gastroenterologist and surgeon. The phrase “*No man is an island*” by John Donne (1572–1631) emphasizes the importance of consensus between specialists and should always be in the minds of those managing patients with biliopancreatic diseases. 

*Fauze Maluf-Filho* *Fauze Maluf-Filho* *Jose G. de la Mora Levy* *Jose G. de la Mora Levy* *Carlos G. Micames* *Carlos G. Micames*

